# Precarious employment is a risk factor for poor mental health in young individuals in Sweden: a cohort study with multiple follow-ups

**DOI:** 10.1186/s12889-016-3358-5

**Published:** 2016-08-02

**Authors:** Catarina Canivet, Theo Bodin, Maria Emmelin, Susanna Toivanen, Mahnaz Moghaddassi, Per-Olof Östergren

**Affiliations:** 1 Division of Social Medicine and Global Health, Department of Clinical Sciences Malmö, Lund University, SE–205 02 Malmö, Sweden; 2 Division of Occupational and Environmental Medicine, Lund University, SE-221 85 Lund, Sweden; 3 Institute of Environmental Medicine, Karolinska Institutet, SE-171 77 Stockholm, Sweden; 4 CHESS (Centre for Health Equity Studies), Sveavägen 160, SE-106 91 Stockholm, Sweden

**Keywords:** Longitudinal studies, Mental health, Unemployment, Stress, Psychological, Young adult, Employment

## Abstract

**Background:**

The globalisation of the economy and the labour markets has resulted in a growing proportion of individuals who find themselves in a precarious labour market situation, especially among the young. This pertains also to the Nordic countries, despite their characterisation as well developed welfare states with active labour market policies. This should be viewed against the background of a number of studies, which have shown that several aspects of precarious employment are detrimental to mental health. However, longitudinal studies from the Nordic region that examine the impact of precarious labour market conditions on mental health in young individuals are currently lacking. The present study aims to examine this impact in a general cohort of Swedish young people.

**Methods:**

Postal questionnaires were sent out in 1999/2000 to a stratified random sample of the Scania population, Sweden; the response rate was 58 %. All of those who responded at baseline were invited to follow-ups after 5 and 10 years. Employment precariousness was determined based on detailed questions about present employment, previous unemployment, and self-rated risk of future unemployment. Mental health was assessed by GHQ-12. For this study individuals in the age range of 18–34 years at baseline, who were active in the labour market (employed or seeking job) and had submitted complete data from 1999/2000, 2005, and 2010 on employment precariousness and mental health status, were selected (*N* = 1135).

**Results:**

Forty-two percent of the participants had a precarious employment situation at baseline. Labour market trajectories that included precarious employment in 1999/2000 or 2005 predicted poor mental health in 2010: the incidence ratio ratio was 1.4 (95 % CI: 1.1–2.0) when excluding all individuals with mental health problems at baseline and adjusting for age, gender, social support, social capital, and economic difficulties in childhood. The population attributable fraction regarding poor mental health in the studied age group was 18 %.

**Conclusions:**

This study supported the hypothesis that precarious employment should be regarded as an important social determinant for subsequent development of mental health problems in previously mentally healthy young people.

**Electronic supplementary material:**

The online version of this article (doi:10.1186/s12889-016-3358-5) contains supplementary material, which is available to authorized users.

## Background

Due to several decades of globalisation combined with predominantly neoliberal economic policies, long-term employment contracts and a high level of job security is no longer the dominant form of labour market relation in a global perspective. Instead, short-term contracts, involuntary part time employment, employment through ‘staff-for-hire’ enterprises, and shorter or longer periods of unemployment have become increasingly common [1]. Already in 1998, in a report from the European Foundation for the Improvement of Working and Living Conditions (Eurofound; published online in 2012), it was shown that fixed-term and temporary contracts were associated with low incomes, short working hours, and poor working conditions [2]. The temporary employment rate in the 27 EU countries has since climbed from 11.2 % in 2001 to 12.8 % in 2012. The corresponding figures for Sweden are 13.8 % and 14.7 % [3].

Several aspects of precarious employment have been shown to be detrimental to mental health in a number of population surveys. A recent review by Benach et al. found studies supporting a linkage between mental health problems and major organisational restructuring and downsizing, perceived job insecurity, and temporary employment [4]. New research indicates that the latest financial crisis of 2008 led to an increase in poor mental health among the European population [5]. Since there is a strong likelihood of bidirectional causality between labour market factors on the one hand and mental health on the other [6, 7], longitudinal studies are of paramount importance in order to disentangle causal directions.

The impact on health of labour market relations among young individuals has been studied longitudinally in the Northern Swedish Cohort, which was established in 1981 [8]. Unemployment was associated with subsequent mental health problems [9], and similar results have been found in studies on young cohorts in Britain [10] and US [11]. In another longitudinal study, from New Zeeland, the association between unemployment and poor mental health disappeared after adjustment for confounders and reverse causality [6]. It is sometimes assumed that persons early in their working life may be able, later on, to overcome economic and health setbacks associated with unemployment. This was one of the working hypotheses in a recent large meta-analysis covering studies on unemployment and mental health. However, contrary to expectations, the effect of unemployment was larger among persons of young (and old) age versus those in middle-age [12]. In line with this finding are studies demonstrating a ‘scarring’ effect of unemployment in youth, i.e. that the difference in mental health between those who have been exposed to labour market insecurity, versus those who have not, tends to become more pronounced over time [13, 14].

It is often argued that well developed welfare states with active labour market policies can buffer the negative effects of a precarious labour market [15]. In a recent Swedish study, it was found that programs directed at young persons at risk of labour market exclusion led to a positive mental health development later in life, compared to those who experienced unemployment during the same time period [16].

Young persons are overrepresented both among those unemployed and among those in temporary employment [17, 18]. In Sweden, the proportion of persons aged 20–24 who were not in education, employment, or training (NEET) was 9.9 % as of 2014 [19]. In 2015, temporary contracts were held by 33% of all women aged 20–34 active in the labour market, and by 27% of the men, while the corresponding figures for the whole labour force were 16% and 14% [20]. Moreover, prevalence figures do not reveal the true proportion of the young population that is exposed to unemployment or a precarious work situation over time, and the number affected over a longer period of time could be expected to be considerably greater.

There are few longitudinal studies from the Nordic countries, as well as from an international perspective, that examine the situation of young people entering the labour market and that use data sets collected after year 2000. The aim of the present study was thus to investigate the associations between precarious employment situations and mental health later in life among young adults aged 18–34 in a Swedish setting, using a longitudinal study design.

## Methods

### Study population

The Scania Public Health Cohort, Sweden, was established in 1999/2000; for details see Carlsson et al. [21]. In short, 58 % (*N* = 13604) of a stratified random sample of the Scania population, 18–80 years of age, responded to a postal questionnaire, which was sent out again in 2005 and 2010 to all responders. Out of those who were 18–34 years old at baseline (*N* = 3420), 1802 participated also in the two follow-up surveys. The cohort selected for the present study consists of those (*N* = 1135) who had complete data from 1999/2000, 2005, and 2010, both on employment precariousness and the outcome measure, i.e. mental health status, and who, according to their inquiry responses, were considered to be part of, or close to the labour market (see below).

### Outcome variable – poor mental health

Mental health was measured at all three time points using the 12-item version of the General Health Questionnaire (GHQ-12). We used the 0-0-1-1 scoring method recommended by the creators of the instrument (range 0–12), with poor mental health or ‘GHQ-caseness’ defined as a scoring of 2 or higher [22].

### Precarious employment

At the time of the baseline survey, a validated measure of employment precariousness was not available. However, the health effects of precarious employment have been described using several research approaches, included focusing on a) temporary vs. permanent employment, b) perceived job insecurity, and c) major organisational restructuring and downsizing [4]. The construct of work history has also been proposed to constitute a useful element in defining precariousness [23], and unemployment at a young age was recently shown to predict later unemployment during 15 years of follow-up [24]. We chose to create a dichotomous variable – non-precarious (NP) vs. precarious employment (PE) – based on a combination of data on present unemployment, previous unemployment, temporary vs. permanent employment, and perceived job insecurity. The questions asked were as follows:

- Which of the following applies best to you at the present moment (response alternatives: working for a living, student, no work outside home, disability pension/long term sick leave, unemployed)?
- If employed, which are the terms of your contract? Response alternatives were ‘permanent’, ‘substitute’, ‘fixed term’, and ‘on demand’. Several alternatives could be chosen, and the responses were dichotomised into ‘permanent (only)’ and ‘contingent’.
- Have you been involuntarily unemployed at some point during the past three years (no, yes)?
- How do you rate your own risk of involuntary unemployment within the coming year? (high, moderate, low, none, ‘I do not wish to work a year from now’).

Those on disability pension/long tem sick leave were excluded from the cohort, as well as those who answered that they did not wish to work a year from now, since they were not considered to be relevant for the target group in focus of this research, i.e. young persons active in the labour market.

Those categorised as NP were those with permanent work and no or low self-rated risk of future unemployment and those with contingent work and no self-rated risk of future unemployment and no previous unemployment. Those categorised as PE were others with contingent work, others with previous unemployment, all those with moderate to high self-rated risk of future unemployment, and all presently unemployed.

### Other measures

The following measures were also assessed (for details about inquiry questions and response alternatives, see Additional file 1): Age (continuous variable), country of origin, marital status, education level at baseline, economic difficulties, alcohol consumption [25] physical activity [26], emotional and instrumental support, social participation [27], and social anchorage with neighbourhood.

We used economic difficulties in the family while growing up as a proxy for socioeconomic position. The response alternatives to this question were ‘no, none worth mentioning’, ‘yes, slight or relatively short periods with economic difficulties’, and ‘yes, severe and/or longer periods with economic difficulties’. The dichotomisation was performed at the level of ‘no’ versus the other two.

The study was approved by the Research Ethics Committee of Lund University (1999–99; 2005–471, and 2010–392).

### Statistical methods

The relationships between background factors and poor mental health in 2010 are presented as percentages and age-adjusted incidence rate ratios (IRR) which is a good estimate of relative risks, using a modified Poisson regression model with robust standard errors [28].

In an attempt to clarify possible causal mechanisms, several analyses were performed in which all participants with poor mental health at baseline (*N* = 349) were excluded. In Table 4, PE in 1999/2000 or 2005 is thus tested against poor mental health in 2010 with the stepwise addition of possible confounders, measured at baseline. Since emotional and instrumental support at baseline correlated strongly (*r* = 0.5 in this group), a composite variable was used (no exposure versus exposed to either or both) in the multivariate analyses. The population attributable fraction (i.e. the proportion of disease cases over a specified time that would be prevented following elimination of the exposures, assuming the exposures are causal) [29] for PE in poor mental health was calculated using the formula: PAF = pd × (RR − 1)/RR [29], where pd was the proportion of cases exposed to PE, and the RR was the IRR for poor mental health, after full adjustment with the same covariates as in Table 3. Tests for effect modification were performed for economic difficulties in childhood, gender, education level, and age groups. This was done by creating dummy variables and synergy indexes, as proposed by Rothman [30].

Two standard statistical analysis programs were used, i.e. SPSS version 22.0 and Stata version 12.

## Results

In the original study population (*N* = 3420) of young persons participating in the baseline inquiry in 1999/2000, there were 1802 (53 %) who also took part in both of the surveys in 2005 and 2010. Those who did not take part in both surveys (*N* = 1618, 47 %) were to a higher degree younger (mean age 26 vs. 27), of male gender (50 vs 41 %), of low education level (65 vs. 51 %), born outside Sweden (13 vs 9 %), in economic difficulties (38 vs. 27 %), affirming poor self-rated health (27 vs 23 %), and in a PE situation (52 vs 43 %). However, there was no difference in mental health status; thus, 33 vs. 31 % had a GHQ-score indicating poor mental health (Pearson chi-square 1.4; *p* = 0.24). (The numbers missing in the above-mentioned analyses varied from 0 to 81, except for PE: missing = 781, whereof 410 from the group of 1618 non-respondents).

Both among respondents and non-respondents, the baseline cross-sectional association between PE and poor mental health was strong, with a Pearson chi square value in respondents and non-respondents of 56.7 and 32.7, respectively (both *p* < 0.001).

Thereafter, in the group of participants in all three surveys (*N* = 1802), those constituting the final study cohort (*N* = 1135, with complete data, see Methods) were compared to those with lacking data (*N* = 667). There were no differences regarding gender, country of birth, economic difficulties, self-rated health, or mental health. However, a low education level was more common among those with lacking data (56 vs. 49 %).

Data on employment precariousness is presented in Table 1. In this cohort, the proportion of participants in a PE situation decreased during the 10-year study period, from 42.1 % in 1999/2000 to 22.9 % in 2010. The individual trajectories are seen in Table 2. The most common trajectory (40.2 %) was having a NP situation at all measurement points. The next most common trajectory was PE – NP – NP (17.9 %), whereas 11.0 % had a PE situation at all measurement points. At baseline, 62 persons were unemployed (5.5 %) and at follow-up 53 persons, but only seven had been unemployed at both time points (not shown in Table).

**Table 1:** Categorisation of employment precariousness and prevalences. Employment precariousness status in 1999/2000, 2005, and 2010 of 1135 young participants from the Scania Public Health Cohort

Characteristics of preliminary categories	Final categories	1999/2000		2005		2010	
		*n*	%	*n*	%	*n*	%
Permanent work	Non-precarious employment (NP)	575	50.7	739	65.1	859	75.7
+ no or low self-rated risk of future unemployment (regardless of previous unemployment)							
Contingent work	Non-precarious employment (NP)	82	7.2	27	2.4	16	1.4
+ no self-rated risk of future unemployment + no previous unemployment							
All others with contingent work AND/OR moderate to high self-rated risk of future unemployment AND/OR previous unemployment	Precarious employment (PE)	416	36.6	306	26.9	207	18.2
Unemployed	Precarious employment (PE)	62	5.5	63	5.6	53	4.7
		1135	100	1135	100	1135	100

**Table 2:** Trajectories of employment status

1999/2000 – 2005 – 2010	ns	%
NP^a^ – NP – NP	456	40.2
PE^b^ – NP – NP	203	17.9
PE – PE – NP	125	11.0
PE – PE – PE	105	9.3
NP – PE – NP	91	8.0
NP – NP – PE	62	5.5
NP – PE – PE	48	4.2
PE – NP – PE	45	4.0
	1135	100.0

^a^ *NP* Non-precarious employment

^b^ *PE* Precarious employment

The proportion of individuals with poor mental health was 31 % in 1999/2000, 31 % in 2005, and 26.5 % in 2010.

Table 3 shows that PE in 1999/2000 or 2005 was associated with poor mental health at follow-up in 2010. The age-adjusted IRR was 2.0 (95 % CI (confidence interval): 1.6–2.6) for those exposed to PE in both 1999/2000 and 2005.

**Table 3:** Sociodemographic characteristics and employment precariousness in relation to poor mental health. Unless stated otherwise, figures are from baseline in year 1999/2000, with incidence rate ratios (IRR) and 95 % confidence intervals (CI). The outcome is poor mental health, defined as ‘GHQ-caseness’ at follow-up in 2010

Variable (numbers missing)		N/valid %	% Poor mental health in 2010	IRR	95 % CI
Age	18–23	265/23.3	26.4	1.0	
	24–29	420/37.0	28.8	1.1	0.8 – 1.4
	30–34	450/39.6	24.4	0.9	0.7 – 1.2
	Total	1135	26.5		
Gender	Male	461/40.6	20.8	1.0	
	Female	674/59.4	30.6	1.5	1.2 – 1.8
Country of birth (8)	Sweden	1038/92.1	25.8	1.0	
	Other	89/7.9	34.8	1.4	1.003 – 1.8
Married or cohabiting (17)	Yes	686/61.4	25.2	1.0	
	No	432/38.6	28.7	1.1	0.9 – 1.4
Education level (14)	≥13 y	574/51.2	26.0	1.0	
	≤ 12 y	547/48.8	27.1	1.0	0.8 – 1.3
Economic difficulties (17)	No	824/73.7	22.7	1.0	
	Yes	294/26.7	37.4	1.6	1.4 – 2.0
Economic difficulties in childhood (reported at baseline) (8)	No	816/72.4	25.0	1.0	
	Yes	311/27.6	31.2	1.2	1.01 – 1.5 1.6
Economic difficulties in 2010 (31)	No	939/85.1	23.1	1.0	
	Yes	165/14.9	46.7	2.0	1.6 – 2.5
Risky alcohol consumption (27)	No	1014/91.5	25.4	1.0	
	Yes	94/8.5	33.0	1.3	0.94 – 1.7
Low physical activity (26)	No	958/86.4	26.0	1.0	
	Yes	151/13.6	26.5	1.0	0.8 – 1.4
Low emotional support (8)	No	829/73.6	22.8	1.0	
	Yes	298/26.4	35.9	1.6	1.3 – 1.9
Low instrumental support (4)	No	930/82.2	23.7	1.0	
	Yes	201/17.8	39.3	1.7	1.4 – 2.0
Low social participation	No	960/84.6	24.8	1.0	
	Yes	175/15.4	36.0	1.5	1.2 – 1.8
Low social anchorage with neighbourhood (12)	No	708/63.0	24.3	1.0	
	Yes	415/7.0	30.8	1.3	1.04 – 1.5
Poor mental health 1999/2000 – 2005	No – No	610/53.7	15.9	1.0	
	Yes – No	175/15.4	28.0	1.8	1.3 – 2.4
	No – Yes	176/15.5	31.8	2.0	1.5 – 2.7
	Yes – Yes	174/15.3	56.9	3.6	2.9 – 4.5
Precarious employment 1999/2000 – 2005	No – No	518/45.6	18.9	1.0	
	Yes – No	248/21.9	29.8	1.6	1.2 – 2.1
	No – Yes	139/12.2	30.9	1.6	1.2 – 2.2
	Yes – Yes	230/20.3	37.4	2.0	1.6 – 2.6
Precarious employment 1999/2000 – 2005, dichotomised	No	518/45.6	18.9	1.0	
	Yes	617/54.4	32.9	1.8	1.4 – 2.2

As can be seen in Table 4, excluding all persons with poor mental health at baseline, PE in 1999/2000 or 2005 was associated with poor mental health at follow-up also in the fully adjusted model; the IRR was 1.4 (95 % CI: 1.1–2.0).

**Table 4:** Precarious employment in 1999/2000 or 2005 and mental health at follow-up. Age-adjusted incidence rate ratios with 95 % confidence intervals of poor mental health, defined as ‘GHQ-caseness’ in 2010, in relation to precarious employment in 1999/2000 or 2005, with forward stepwise addition of potential confounding factors, calculated in a cohort of 347 men and 439 women from the Scania Public Health Cohort who were 18–34 years old and mentally healthy at baseline

		Model 1 (age-adjusted)		Model 2 = Model 1		Model 3 = Model 2		Model 4 = Model 3		Model 5 = Model 4	
				+		+		+		+	
				Gender		Low emotional and/or low instrumental support		Low social participation & neighbourhood anchorage		Economic difficulties in childhood	
Precarious employment 1999/2000 – 2005	Yes vs no	1.6	1.2 – 2.2	1.6	1.2 – 2.1	1.5	1.1 – 2.1	1.5	1.1 – 2.0	1.4	1.1 – 2.0
Gender	Male vs female			1.3	0.99 – 1.8	1.4	1.03 – 1.9	1.4	1.02 – 1.9	1.4	1.01 – 1.8
Low emotional and/or low instrumental support	Yes vs no					1.3	0.9 – 1.7	1.1	0.8 – 1.6	1.1	0.8 – 1.5
Low social participation	Yes vs no							1.5	1.1 – 2.1	1.5	1.1 – 2.1
Low social anchorage with neighbourhood	Yes vs no							1.3	0.9 – 1.7	1.3	0.9 – 1.7
Economic difficulties in childhood	Yes vs no									1.2	0.9 – 1.7

Among those mentally healthy at baseline, and after adjustment with the same variables as in Table 4, the population attributable fraction for PE in 1999/2000 or 2005 and poor mental health at follow-up was 18 %.

No effect modification on the relationship between PE and mental health was found for economic difficulties in childhood, age groups, gender, or education level (results not shown).

## Discussion

### Principal findings

This study found that among previously mentally healthy young adults in southern Sweden, labour market trajectories including a precarious employment situation in 1999/2000 or in 2005 was a predictor for poor mental health in 2010. The IRR was 1.4 (95 % CI: 1.1–2.0) after adjustment for age, gender, emotional and instrumental support, social participation and neighbourhood anchorage, and economic difficulties in childhood. The calculated population attributable fraction for a precarious employment situation regarding poor mental health in this group was 18 %.

### Findings in relation to other studies and possible mechanisms

Mental health problems have become increasingly common among young people during the last decades, and particularly so in Sweden. In 2011, seven percent of men and 10 % of women aged 16–24 years old had been in touch with a psychiatric clinic or used psychotropic drugs [31]. It has been suggested that recent changes in the labour market, increasing the risk of experiencing precariousness, have contributed to the rapidly deteriorating trend in mental health among young people [32].

In a meta-analysis from 2002, covering international studies on job insecurity and its consequences, a relatively strong effect size for mental health was found (mean correlation: −0.237) [33]. Several recent cross-sectional [23, 34] and longitudinal [35–37] studies also support the relationship found between job insecurity and mental health problems. Benach et al. suggest that one pathway from job insecurity to adverse health outcome may consist of stress response to sustained uncertainty, unpredictability, and lack of control over the future [4].

The current study primarily focuses on aspects of employment insecurity in the definition of PE used here. Another link between PE and poor mental health may consist of poorer working conditions for persons with PE. These may include poorer supervision, inadequate training, exposure to higher risk tasks, lack of workplace voice, economic and reward pressures, disorganisation at the workplace, and regulatory failure [38].

In one study, insecure employment negatively affected the likelihood of getting married and having children, which could be a mediating factor for poorer mental health in young people [39].

Fear of the stigmatisation connected to a precarious labour market may also play a part. In a recent qualitative study, indications of negative stereotyping about ‘unemployed persons’ were found among nurses working in a healthcare program for job seekers [40]. On the other hand, it could be argued that since precarious employment is steadily increasing, and in particular among young people, the potential stigmatisation of not having a stable employment may become less pronounced with time.

This study was performed in Sweden. The degree of health impairment of individuals with precarious employment was least in Scandinavian settings in one comparative study [15]. Scandinavian societies have been characterised as egalitarian welfare states, with effective collective bargaining institutions, lifelong job training, and generous unemployment schemes, all of which may contribute to a buffering effect. Therefore, it could be hypothesised that a similar study performed elsewhere might have shown even stronger associations between precarious employment and poor mental health.

However, a study performed in Sweden today might yield greater associations compared with previous decades, since ‘Scandinavian welfare’ is not a constant. A recent Swedish study showed that mental distress among women increased between 2006 and 2010, and more so among groups outside the labour market [41]. The authors suggested that one of the reasons might be the considerable modifications, e.g. stricter eligibility criteria and lower benefit levels, which have been implemented by the Swedish social insurance system during the last decade.

Moreover, the global trend towards precarious labour market relations seems different than previous cyclic unemployment situations. Not only have the past decades of neoliberal politics, with general deregulation and privatisations, led to a shift in power relations characterised by a markedly increased influence of employers vis-à-vis workers [42, 43], but also, a broad range of changes have occurred regarding the individual’s relation to society and the capacity of the welfare state to buffer the negative impacts among those exposed to this situation. The British sociologist Guy Standing has developed the concept of precarity, and he states: *‘This is not just a matter of having insecure employment, of being in jobs of limited duration and with minimal labour protection, although all this is widespread. It is being in a status that offers no sense of career, no sense of secure occupational identity and few, if any, entitlements to the state and enterprise benefits that several generations of those who saw themselves as belonging to the industrial proletariat or the salariat* (non-manual employees with secure employment) *had come to expect as their due.’* [44] p. 24. Standing also suggests that it is of major public health interest to assess the potential negative health impacts of such living conditions for the increasing numbers of persons living in this *‘class-in-the-making’* [44].

### Methodological considerations

The study was designed to investigate a causal relation between a PE situation and mental health problems in young persons. We thus used a model excluding all persons with poor mental health at baseline and followed the trajectory over two subsequent follow-up occasions, which reduces the risk of reversed causality.

Other strengths of our study include the adjustment for a substantial number of potential confounders and the recruitment from a large random general population sample. However, a first selection took place in the very establishment of The Scania Public Health Cohort, since the response rate was 58 % for women 18–30 years old and 45 % for men in the same age range [45]. Moreover, our study group (participating in all three surveys) differed from the original cohort regarding several characteristics (education level, country of origin, economic difficulties, and self-rated health), which makes it reasonable to assume [2] that PE may have been more common among non-respondents. This was indeed the case for those non-respondents who supplied this information at baseline (1208 out of 1618), where 52 % reported PE. Since the baseline association between exposure and outcome was similar in both groups, we conclude that selection bias in our final study sample may have resulted in an underestimation of the association at follow-up between PE and poor mental health in the general population of young people.

Our measure of PE is based on extensive information on relevant indicators but has not been validated previously. It is highly probable that a multifaceted and continuous measure of precarity such as the comprehensive EPRES (The Employment Precariousness Scale) [46] would have resulted in a more accurate description and conceivably a discernible dose–response association between exposure and outcome.

Moreover, the ‘forced’ dichotomisation of our instrument may have led to some misclassified cases. For instance, we chose to classify those with ‘contingent work but no previous unemployment and no self-rated risk of future unemployment’ as NP cases. However, and perhaps particularly in this population of young people, it may be argued that they are correctly classified. As stated in a recent EU-commission report on new forms of employment, aspects of the ‘flexible’ labour market may be utilised as a positive choice by some individuals who have competitive characteristics regarding the labour market, and who do not risk unemployment despite the lack of a long-term contract [47]. This phenomenon is discussed also in Guy Standing’s seminal book ‘The precariat – the new dangerous class’ [44].

In this context it should be noted that we chose to categorise unemployment as one form of PE. This is in contrast to the EPRES, where persons without employment contracts were excluded [46], and also in contrast to the ‘peripheral employment score’ [48]. In the latter study, exposure to peripheral employment was positively related to psychological distress, but adjustment for unemployment attenuated the association. Our model, in which unemployment is seen as an extreme form of precarious employment, could thus be debated. However, the fact that only 7 out of 62 unemployed persons remained so after ten years could indicate that there is a considerable mobility in and out of this category, and excluding these very persons, as was done in the EPRES study, would restrict the study findings to those concerning persons with a less precarious situation.

Changes in precarious employment status may have taken place between the measurement of exposure in 1999/2000 to 2005 and the measure of the outcome in 2010. In particular, the economic recession in 2008 may have led to a number of persons being misclassified as NP, which could have biased the results towards the null, and thus led to an underestimation of the associations. On the other hand, only 23 % of the participants were in a PE situation at follow-up, versus 42 % in 1999/2000, which partly may be explained by the fact that the participants had had 10 years to establish themselves on the labour market.

The determination of a cut-off point for the GHQ-12 test is a trade-off between sensitivity and specificity. Since the mean values in the population were below 1.85 (1.22 in 1999/2000 and 1.01 in 2010), we chose the threshold of 1/2, as advocated by the creator of the test [22].

There were several reasons for choosing financial difficulties during childhood as a proxy for socioeconomic status in the multivariate analyses. A large proportion (*N* = 186) of the participants were students, i.e. thus with uncertain present and future socioeconomic position. Furthermore, since the focus of the study was on precarious employment, we firstly considered it essential to identify and categorise all unemployed persons, and secondly, we expected that being unemployed would influence and overrule any socioeconomic position to the point where a classification of socioeconomic status according to job description would become meaningless. Lastly, socioeconomic position according to job description showed no correlation with the outcome. On the other hand, it is reasonable to assume that parental social position in the form of background economic and cultural resources must have influenced these young persons’ present circumstances, both in terms of risk for a precarious employment situation and for poor mental health.

## Conclusions

In Sweden, precarious employment has increased since the Nineties and accelerated after 2008. Cross-sectional studies suggesting a relationship between precarious employment and poor mental health are abundant, but the causal direction could be debatable in this type of studies. The present investigation is one of the first cohort studies to show that a precarious employment situation is an important risk factor for subsequent development of mental health problems among previously mentally healthy young adults. Moreover, due to the probable influence of selection bias in our final study sample, the current association found between precarious employment and poor mental health should be regarded as an underestimate. Therefore, further research is needed to clarify the nature of the association and the underlying mechanisms. The estimated population attributable fraction of 18 % in the group of mentally healthy young people in this study should serve as a wake-up call for politicians and policy makers.

## Abbreviations

CI, confidence interval; EPRES, the employment precariousness scale; GHQ, general health questionnaire; IRR, incidence rate ratio; NP, non-precarious employment; PAF, population attributable fraction; PE, precarious employment

## Additional file

Additional file 1: Other variables and corresponding questions in the questionnaire, i.e. a list with those variables that were used in the analyses, and mentioned, but not described in detail, in the manuscript. (DOCX 16 kb)
