# Supplementary material for: Precarious employment is a risk factor for poor mental health in young individuals in Sweden: a cohort study with multiple follow-ups
Source: BMC Public Health. 2016 Aug 2;16:687. doi: 10.1186/s12889-016-3358-5 (PMC4969669; doi:10.1186/s12889-016-3358-5)
Supplement: Additional file 1: — Other variables and corresponding questions in the questionnaire, i.e. a list with those variables that were used in the analyses, and mentioned, but not described in detail, in the manuscript. (DOCX 16 kb) [file 12889_2016_3358_MOESM1_ESM.docx]

**Appendix.**  Other variables and corresponding questions in the inquiry.

| **Variable** | **Question in inquiry** |
| --- | --- |
| Country of origin | Born in Sweden/Not born in Sweden |
| Marital status | Married or cohabiting/Not married or cohabiting |
| Education level at baseline | Self-reported total years of formal education; dichotomised as ≤ 12 years and ≥ 13 years |
| Economic difficulties in the family while growing up | ‘Did you have economic difficulties in the family while growing up?’ Answer alternatives were ‘no, none worth mentioning’, ‘yes, slight or relatively short periods with economic difficulties’, and ‘yes, severe and/or longer periods with economic difficulties’ (dichotomisation between ‘no’ and the other two alternatives). |
| Economic difficulties, at baseline and at follow-up | a) ‘How often during the last 12 months have you had problems paying your bills?’ and  b) ‘In case of an unforeseen emergency, could you raise 12000 SEK (approximately 1300 EUR) in a week’s time?’  Having economic difficulties was defined as affirming ‘about half of the months’ (or more often) on a) or ‘no’ on b). |
| Alcohol consumption | Gender-adjusted amounts of alcohol use during the previous month were categorised by the quantity–frequency method and dichotomised into ‘risky alcohol consumption’, ‘yes’ versus ‘no’. |
| Physical activity | ‘How often are you physically active or perform exercise during you leisure time? Excluding domestic work’. Answer alternatives were ‘sedentary’, ‘moderate physical activity’, ‘regular exercise’, and ‘regular advanced exercise (dichotomisation between ‘sedentary’ and ‘moderate physical activity’. |
| Emotional support | ‘Do you feel that you have one or several persons who can give you solid personal support in order to handle life’s stress and problems?’ (dichotomised at the level of ‘Yes, definitely’ versus all other answer alternatives) |
| Instrumental support | ‘Could you get help from one or several persons in case of sickness or practical problems (borrow things, help with repairs, help to formulate a document, get advice or information)?’ (dichotomised at the level of ‘Yes, definitely’ versus all other answer alternatives). |
| Social participation | ‘Have you attended *…(various formal and informal social activities)*… during the past year?’ Having attended ≤4 of 13 such activities was defined as low social participation. |
| Social anchorage with neighbourhood | ‘Do you have a sense of kinship and of being rooted in your neighbourhood’? Answers were dichotomised between ‘not at all’ and ‘not particularly’ on the one hand, and ‘to some extent’, and ‘to a high degree’ on the other. |
